# Robot-assisted radical nephroureterectomy using the KangDuo Surgical Robot-01 System versus the da Vinci System: a multicenter prospective randomized controlled trial

**DOI:** 10.1590/S1677-5538.IBJU.2024.0230

**Published:** 2024-08-10

**Authors:** Zhongyuan Zhang, Zhenyu Li, Weifeng Xu, Xuan Wang, Shengcai Zhu, Jie Dong, Xiaojun Tian, Wei Zuo, Qi Tang, Zhihua Li, Kunlin Yang, Xiaoqiang Xue, Yingjie Li, Hongxian Zhang, Qiming Zhang, Silu Chen, Zhaoheng Jin, Xuesong Li, Zhigang Ji, Lulin Ma, Ming Liu

**Affiliations:** 1 Peking University Institute of Urology Peking University First Hospital Beijing China Department of Urology, Peking University First Hospital, Institute of Urology, Peking University, National Urological Cancer Center, Beijing, China; 2 Peking Union Medical College Peking Union Medical College Hospital Department of Urology Beijing China Department of Urology, Peking Union Medical College Hospital, Peking Union Medical College, Chinese Academy of Medical Sciences, Beijing, China; 3 Institute of Geriatric Medicine National Center of Gerontology Beijing Hospital Beijing China Department of Urology, Beijing Hospital, National Center of Gerontology, Institute of Geriatric Medicine, Chinese Academy of Medical Sciences, Beijing, China; 4 Peking University Third Hospital Department of Urology Beijing China Department of Urology, Peking University Third Hospital, Beijing, China

**Keywords:** Robotic Surgical Procedures, Nephroureterectomy, Multicenter Studies as Topic

## Abstract

**Introduction:**

We aim to compare the safety and effectiveness of the KangDuo (KD)-Surgical Robot-01 (KD-SR-01) system and the da Vinci (DV) system for robot-assisted radical nephroureterectomy (RARNU).

**Materials and Methods:**

This multicenter prospective randomized controlled trial was conducted between March 2022 and September 2023. Group 1 included 29 patients undergoing KD-RARNU. Group 2 included 29 patients undergoing DV-RARNU. Patient demographic and clinical characteristics, perioperative data, and follow-up outcomes were collected prospectively and compared between the two groups.

**Results:**

There were no significant differences in patient baseline demographic and preoperative characteristics between the two groups. The success rates in both groups were 100% without conversion to open or laparoscopic surgery or positive surgical margins. No significant difference was observed in docking time [242 (120-951) s vs 253 (62-498) s, P = 0.780], console time [137 (55-290) min vs 105 (62-220) min, P = 0.114], operative time [207 (121-460) min vs 185 (96-305) min, P = 0.091], EBL [50 (10-600) mL vs 50 (10-700) mL, P = 0.507], National Aeronautics and Space Administration Task Load Index scores, and postoperative serum creatinine levels between the two groups. None of the patients showed evidence of distant metastasis, local recurrence, or equipment-related adverse events during the four-week follow-up. One (3.4%) patient in Group 2 experienced postoperative enterovaginal and enterovesical fistulas (Clavien-Dindo grade III).

**Conclusions:**

The KD-SR-01 system is safe and effective for RARNU compared to the DV Si or Xi system. Further randomized controlled studies with larger sample sizes and longer durations are required.

## INTRODUCTION

Upper tract urothelial carcinoma (UTUC) is a relatively uncommon malignancy, accounting for only 5-10% of all urothelial carcinomas (1). The gold standard treatment for localized high-risk UTUC has been radical nephroureterectomy (RNU) via an open approach with bladder cuff excision (BCE). However, due to concerns regarding perioperative morbidity, there has been a growing interest in exploring minimally invasive approaches as alternative treatment options (2–4).

Since its initial documentation by Clayman et al. in 1991 (5), laparoscopic RNU (LSRNU) has demonstrated comparable oncologic outcomes, reduced morbidity and improved perioperative outcomes compared to the open approach (6). Over the past two decades, robot-assisted RNU (RARNU) has also gained attraction, showing satisfactory oncologic outcomes and improved visualization, dexterity and ergonomics (3, 7–10). Although several newly developed robotic surgical systems such as the Revo-I, Senhance and Versius systems have emerged (11–13), the da Vinci (DV) system (Intuitive Surgical, Sunnyvale, CA, USA) remains dominant in the market. Recently, a novel robotic platform called the KangDuo (KD)-Surgical robot-01 (KD-SR-01) (Suzhou KangDuo Robot Co., Ltd., Suzhou, China), has been introduced in China. Preliminary investigations of the KD system have shown excellent performance in pyeloplasty, partial nephrectomy, and radical prostatectomy (14–17). However, no comparative study has yet been conducted to assess the utilization of the KD and DV systems in RARNU.

To our knowledge, this study is the first multicenter prospective randomized controlled trial aiming to compare the safety and effectiveness of the KD system with the DV system in the context of RARNU. We hypothesize that the KD-SR-01 system is safe and effective for RARNU compared to the DV system.

## MATERIALS AND METHODS

### Patient selection

**Figure 1:**
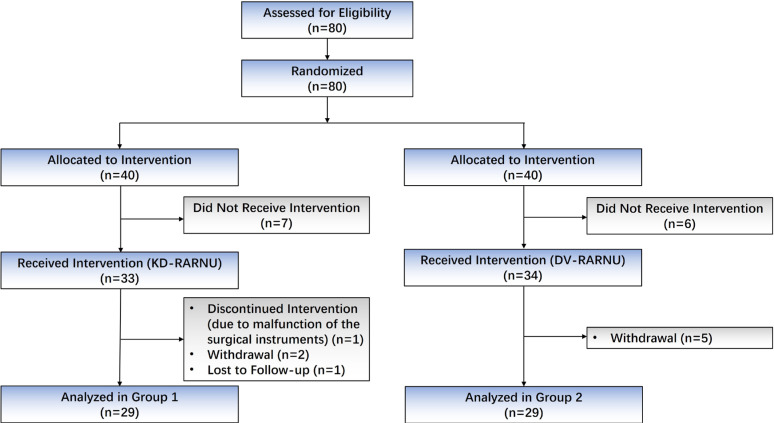
Trial profile.

The protocol of the multicenter randomized controlled trial was approved by the ethics committees of all participating centers. The study was registered at www.chictr.org.cn (ChiCTR2200056672). Between March 2022 and September 2023, patients aged between 18-85 years with a suspicion of ≤T1-3N0M0 UTUC requiring RNU were prospectively included (Figure-1). Exclusion criteria included a history of ipsilateral abdominal surgery, concomitant uncontrolled diseases or urinary tract infection, pregnancy or lactation, relatively high surgical risk or inability to tolerate surgery, and inability or reluctance to cooperate during follow-up. All surgeries were performed by expert surgeons from large tertiary centers with experience with >100 standard robotic surgical procedures, primarily using the DV system. These surgeons had received sufficient training for the KD system, which involved a structured curriculum encompassing comprehensive didactic education, simulation-based training, proctorship under experienced mentors, and hands-on practice in standardized surgical techniques. Prior to the surgery, written informed consent was obtained from all patients, and imaging studies involving chest X-ray, urinary ultrasound, and computed tomography (CT) were performed.

### Randomization and intervention

With randomized block design, the random allocation sequence was generated by the statist using SAS 9.4 and then put in opaque sealed envelopes. The investigator opened an envelope when a new patient entered the study after full communication. Treatment allocation remained masked to both the patients and the investigators until the envelope was opened. The treatment allocation was also masked to the pathologists and individuals who assessed the outcomes for the whole course of study.

**Figure 2:**
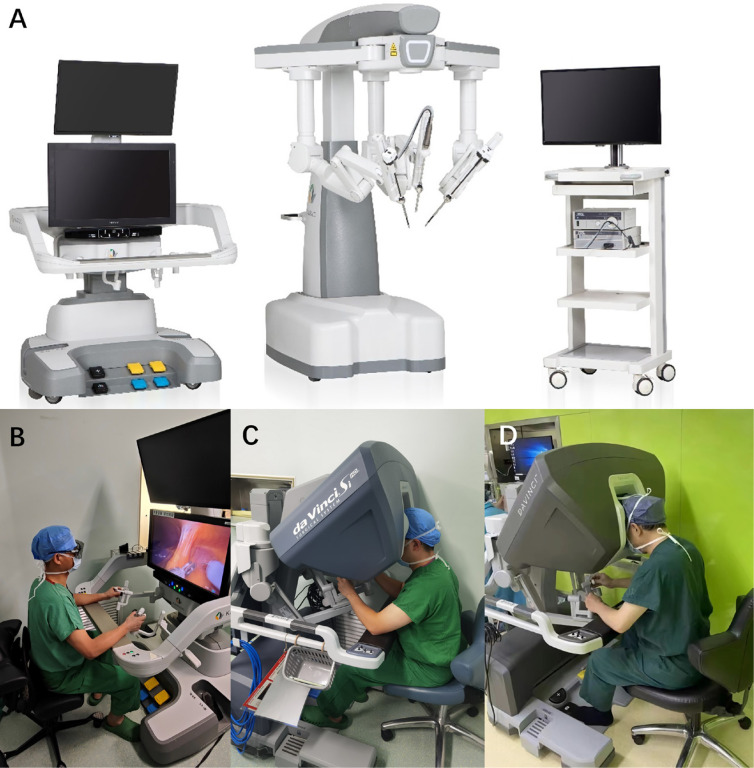
The KD-SR-01 system and the interactions between the surgeon and the consoles of the KD-SR-01 and the DV systems.

Patients were assigned to two groups: Group 1 comprised 29 patients undergoing RARNU with the KD-SR-01 system (KD-RARNU) (Figure-2A), and Group 2 included 29 patients undergoing RARNU with the da Vinci Surgical Si or Xi System (DV-RARNU). The case report form was completed for each patient.

(A) The KD-SR-01 system included the surgeon console, the patient cart, and the vision cart. (B) The surgeon was able to control the open console of the KD-SR-01 system. (C) The surgeon was sitting at the immersive console of the DV Si System. (D) The surgeon was sitting at the immersive console of the DV Xi System.

### Surgical procedures

**Figure 3:**
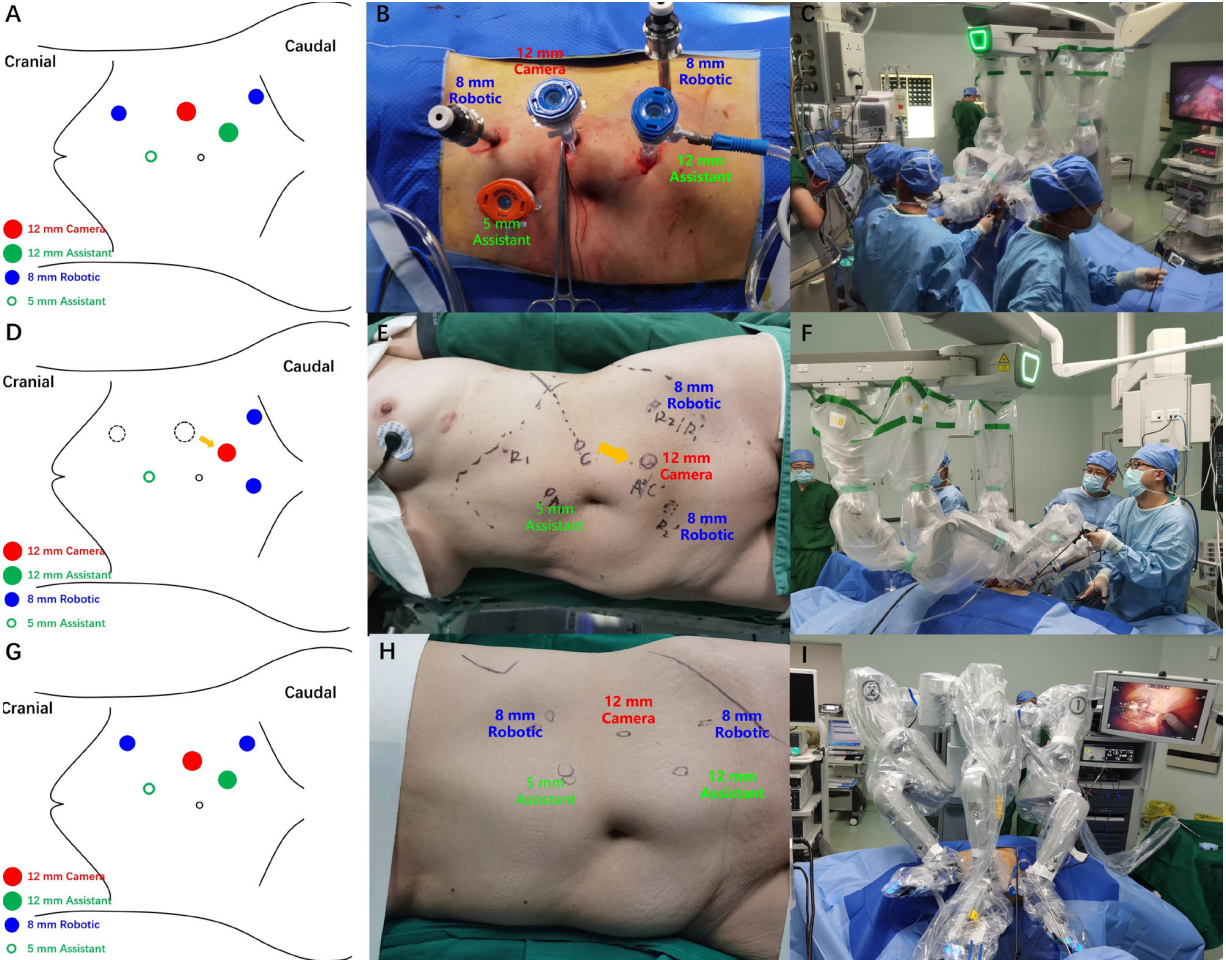
Port placement and robot docking place for KD-RARNU and DV-RARNU.

Under general anesthesia, the patient was positioned in the 45°-60° lateral decubitus position with the lesion side facing upward. The surgeon was sitting in front of the console (Figures 2 B-D), and the assistant was stationed at the patient cart. Three trocars, consisting of two operative trocars and one camera trocar, were used in both robotic systems. Additionally, two assistant trocars were used for suction, retraction, and suture retrieval in both groups (Figure-3). KD-RARNU procedures were performed using either the double-docking technique or the single-docking technique, while DV-RARNU procedures were performed using the single-docking technique only. The double-docking technique necessitated a transition from proximal upper tract dissection to lower tract dissection. The port placement and the robotic docking place were depicted in Figure-3 A-C (first docking) and Figures 3 D-F (second docking). Subsequently, the robotic cart was redocked from a 45° angle entering over the ipsilateral shoulder to a 45° angle entering over the ipsilateral hip. The single-docking technique required the trocar configuration and the robot docking in Figures-3 G-I.

(A and B) The port placement of the first docking. (C) The robotic cart was first docked at a 45° angle entering over the ipsilateral shoulder. (D and E) The port placement of the second docking. (F) The robotic cart was redocked at a 45° angle entering over the ipsilateral hip. (G-I) Single-docking technique for KD-RARNU or DV-RARNU. (G and H) The port placement of the single-docking technique. (I) The robot docking place of the single-docking technique.

**Figure 4:**
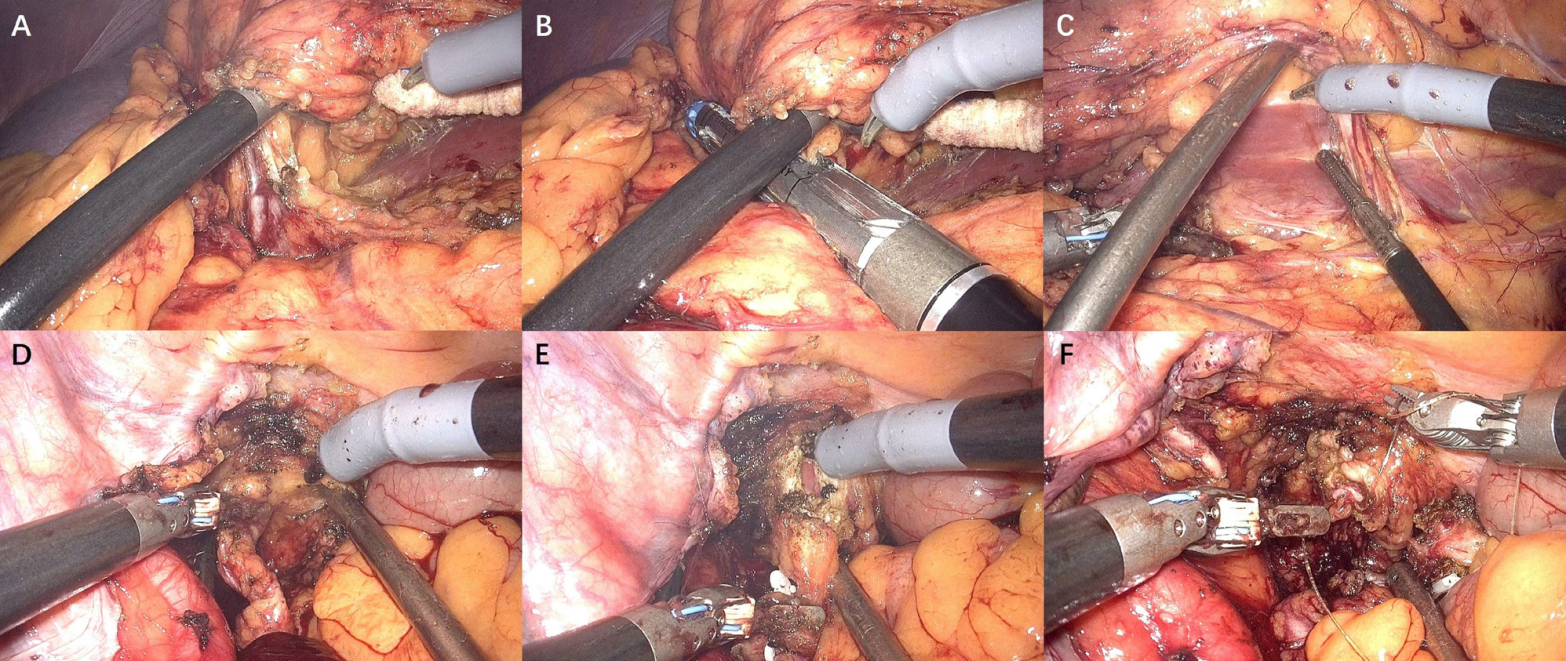
Surgical procedures of RARNU.

Transperitoneal RARNU was performed in both groups using previously described techniques in LSRNU (18, 19). After mobilization of the colon, the renal vein and the renal artery were identified (Figure-4A). The renal hilum was carefully dissected, clipped, and transected using Hem-o-lock or endovascular gastrointestinal anastomosis (Endo-GIA) (Figure-4B). The kidney and the proximal ureter were then dissected (Figure-4C). If necessary, redocking was performed before clipping the ureter distal to the tumor site using Hem-o-lock to prevent tumor seeding. The ureter was meticulously dissected caudally until the ureterovesical junction (Figure-4D). The bottom of the tent-shaped structure was visualized with the retraction of the ureter in the superior and lateral directions. BCE was employed with endoscissors (Figure-4E). Bladder closure could alternatively be achieved by Hem-o-lock clipping or a two-layer running manner using a barbed suture (Figure-4F). Finally, the dissected specimen was extracted en bloc. Lymph node dissection was performed in cases where lymph node metastasis was suspected in the preoperative evaluation or enlarged lymph nodes were found during surgery.

(A) The renal hilum was identified and dissected. (B) The renal artery and the renal vein were transected using Endo-GIA. (C) The kidney and the proximal ureter were dissected. (D) The ureter was dissected carefully caudally until the ureterovesical junction. (E) BCE was performed with endoscissors. (F) The bladder was closed with a two-layer running manner using a barbed suture.

### Data collection and follow-up

Patient demographic and clinical characteristics, perioperative data, and follow-up outcomes were collected prospectively and compared between the two groups. Patient demographic and clinical characteristics included age, gender, body mass index (BMI), laterality, clinical T stage (according to the 2004 World Health Organization grade classification), and preoperative serum creatinine levels. Perioperative data included conversion to open or laparoscopic surgery, docking time, console time, operative time, estimated blood loss (EBL) and the National Aeronautics and Space Administration task load index (NASA-TLX) scores. The docking process was precisely measured from the initiation of the robotic cart to the attachment of the final cannula to the manipulator arm. In cases where the double-docking technique was used, docking time specifically referred to the first-docking time. Console time was defined as the duration spent operating the console to complete the surgical procedures. Subjective evaluation of an estimate of global workload was conducted using the Paper/Pencil Version of the NASA-TLX scores, which was modified from original NASA-TLX continuous rating scale (0-100) to a 20-point scale with the weighting process eliminated and the ratings to simplify the application. Patients were followed up on postoperative day (POD) 1, POD 7, and postoperative week (POW) 4, during which blood and urine tests and physical examinations were conducted. Imaging evaluations such as computed tomography or magnetic resonance imaging were performed on POW 4. The primary endpoint was the success rate of operation determined by the absence of conversion to open or laparoscopic surgery and the presence of negative surgical margins. The secondary endpoint was the postoperative serum creatinine levels. Postoperative complications were categorized according to the Clavien-Dindo system (20).

### Statistical analysis

All statistical analyses were performed using SPSS 27.0 software. The Fisher's exact test or Pearson's chi-square test were used for categorical variables, while the Student t-test or Mann–Whitney U test were used for continuous variables. A probability (P) value of <0.05 was considered significant.

## RESULTS

As shown in Figure-1, a total of 58 patients were included for analysis (n=29 per each group). Patient baseline demographic and preoperative characteristics of the two groups are displayed in Table-1. There were no statistically significant differences regarding age, gender, BMI, laterality, clinical tumor stage, and preoperative serum creatinine levels between the two groups.

**Table 1:** Patient baseline demographic and preoperative characteristics.

Variables		Group 1	Group 2	*P* value
		(n=29)	(n=29)	
**Age, years, mean ± SD**		**63.62±10.34**	**67.31±6.44**	**0.109**
Gender, male/female, n		21/8	14/15	0.060
BMI, kg/m^2^, mean ± SD		25.33±3.32	24.83±3.61	0.582
Laterality, right/left, n		10/19	16/13	0.113
**Clinical tumor stage, n (%)**				0.929
	T1	13 (44.8%)	14 (48.3%)	
	T2	11 (37.9%)	11 (37.9%)	
	T3	5 (17.2%)	4 (13.8%)	
Preoperative serum creatinine levels, μmol/L, mean ± SD		100.90±35.43	101.89±35.53	0.916

SD = standard deviation; BMI = body mass index.

Perioperative data and follow-up outcomes are presented in Table-2. All RARNU procedures were completed without conversion to open or laparoscopic surgery, and positive surgical margins were not noted, resulting in a 100% success rate for both groups. There were no significant differences observed in docking time [242 (120-951) s vs 253 (62-498) s, P = 0.780], console time [137 (55-290) min vs 105 (62-220) min, P = 0.114], operative time [207 (121-460) min vs 185 (96-305) min, P = 0.091], and EBL [50 (10-600) mL vs 50 (10-700) mL, P = 0.507] between the two groups. The global, mental demand, physical demand, temporal demand, performance, effort and frustration of the NASA-TLX scores of Group 1 were 14.38±15.57, 2.59±2.97, 2.97±3.82, 2.93±4.28, 1.38±0.98, 2.66±2.94, and 1.86±1.58, respectively. These scores were comparable to those of Group 2 which were 13.86±13.50, 3.00±3.76, 3.03±3.91, 2.55±3.14, 1.24±0.87, 2.38±2.56, and 1.66±1.42, respectively. Postoperative serum creatinine levels on POD 1 (111.93±38.20 μmol/L vs 115.08±43.67 μmol/L, P = 0.864), POD 7 (116.48±43.23 μmol/L vs 116.92±51.07 μmol/L, p=0.972), and POW 4 (120.70±47.94 μmol/L vs 120.53±58.06 μmol/L, P = 0.990) showed no difference statistically significant between the two groups. No evidence of distant metastasis or local recurrence were found based on imaging evaluation conducted on POW 4.

**Table 2:** Perioperative data and follow-up outcomes.

Variables		Group 1	Group 2	P value
		(n=29)	(n=29)	
Conversion, n		0	0	-
Success rate		100%	100%	-
Docking time. s, median (range)		242 (120-951)	253 (62-498)	0.780
Console time, min, median (range)		137 (55-290)	105 (62-220)	0.114
Operative time, min, median (range)		207 (121-460)	185 (96-305)	0.091
EBL, ml, median (range)		50 (10-600)	50 (10-700)	0.507
**NASA-TLX scores, mean ± SD**				
	Global	14.38±15.57	13.86±13.50	0.893
	Mental demand	2.59±2.97	3.00±3.76	0.644
	Physical demand	2.97±3.82	3.03±3.91	0.946
	Temporal demand	2.93±4.28	2.55±3.14	0.701
	Performance	1.38±0.98	1.24±0.87	0.573
	Effort	2.66±2.94	2.38±2.56	0.705
	Frustration	1.86±1.58	1.66±1.42	0.601
**Serum creatinine levels,** μ**mol/L, mean ± SD**				
	POD 1	111.93±38.20	115.08±43.67	0.864
	POD 7	116.48±43.23	116.92±51.07	0.972
	POW 4	120.70±47.94	120.53±58.06	0.990
Equipment-related adverse events, n		0	0	-
Postoperative complications of Clavien-Dindo grade ≥ III, n (%)		0 (0)	1 (3.4)	1.000

EBL = estimated blood loss; NASA-TLX = National Aeronautics and Space Administration task load index; SD = standard deviation; POD = postoperative day; POW = postoperative week.

No equipment-related adverse events were reported during the follow-up period. No major postoperative complications (Clavien-Dindo grade ≥ III) were noted in Group 1. One (3.4%) patient in Group 2 experienced enterovaginal and enterovesical fistulas (Clavien-Dindo grade III) after surgery, which were repaired by surgical intervention.

## DISCUSSION

RARNU has gained increasing popularity in robotic surgery. The study represents the first multi-center prospective randomized controlled trial to compare the safety and effectiveness of the innovative KD system with the DV system for RARNU. All surgical procedures were successfully completed without open or laparoscopic conversion, and no positive surgical margins were observed, indicating comparable effectiveness profiles. No significant differences were observed in docking time, console time, operative time, EBL, and serum creatinine levels on POD 1, POD 7, and POW 4 between the two groups. Group 1 experienced no equipment-related adverse events or severe (Clavien-Dindo grade ≥ III) postoperative complications, affirming the safety of the KD system.

Regarding the trocar placement and docking techniques, the KD system introduced an additional trocar at the midline of the lower abdomen, and the laparoscopic instruments were shifted between ports during the double-docking procedures, which enabled the transition from the dissociation of the kidney and proximal ureter to the dissociation of the distal ureter and BCE without patient repositioning. In cases where the patient's abdomen was relatively short and the laparoscopic instruments were of sufficient length, the single-docking technique was recommended, especially for DV-RARNU, to alleviate the additional burden of redocking and repositioning. In terms of the BCE technique, a tent-shaped bladder mucosal cuff and intramural ureter could be visualized by retraction in the superior and lateral directions, facilitating en bloc BCE with clear surgical margins both at the base and border of the specimen without urinary spillage (18, 19).

There are several noteworthy features of the KD system. The open surgeon console of the KD system serves to alleviate neck fatigue of the surgeons and enhance communications between surgeons and assistants (16). Furthermore, the KD system is equipped with three suspended arms with synchronous rotation capabilities to accommodate patient position without repositioning. The force sensor technology and the cross-laser design also enhanced the convenience of docking and undocking procedures. In addition, the KD system utilizes a foot clutch, which requires additional training for surgeons familiar with the manual clutch of the DV system to adapt to this new feature. However, the ergonomics of the KD system are comparable to the DV system based on NASA-TLX scores.

Similar to the DV system, the KD system lacks tactile feedback systems, which can be partially compensated by a high-resolution three-dimensional laparoscope for procedures within the deep and confined areas (21). The utilization of single-site technology and remote surgery in the KD system has also been limited. Single-site technology is associated with better cosmetic outcomes (22), and remote surgery eliminates geographical barriers among surgeons, assistants and patients (23). All of these innovations merit further exploration for the advancement of robotic systems, particularly for RARNU.

This study certainly has some limitations. The sample size was relatively small in both groups, which may impact the generalizability of the findings. Additionally, the limited four-week postoperative follow-up period prevents an assessment of long-term oncological outcomes and renal function status after RARNU. Furthermore, although KD-SR-01 is a self-developed Chinese system with a lower estimated cost compared to the DV system, which could potentially benefit more patients by driving prices down, a cost-effective analysis comparing different robotic systems was not conducted.

## CONCLUSIONS

The KD-SR-01 system manifests the safety and effectiveness for RARNU in comparison with the DV Si or Xi system. However, larger-sample and longer-term prospective randomized controlled trials are warranted to assess the oncologic outcomes and renal function status.

## ABBREVIATIONS

BCE: = bladder cuff excision
BMI: = body mass index
CT: = computed tomography
DV: = da Vinci
EBL: = estimated blood loss
Endo-GIA: = endovascular gastrointestinal anastomosis
KD: = KangDuo
KD-SR-01: = KangDuo-Surgical Robot-01
LSRNU: = laparoscopic radical nephroureterectomy
NASA-TLX: = National Aeronautics and Space Administration task load index
POD: = postoperative day
POW: = postoperative week
RARNU: = robot-assisted radical nephroureterectomy
RNU: = radical nephroureterectomy
UTUC: = upper tract urothelial carcinoma

## DATA AVAILABILITY STATEMENT

The data that support the findings of this study are available from the corresponding author upon reasonable request.
